# Validation of dynamic three-dimensional whole heart magnetic resonance myocardial perfusion imaging at 3.0 Tesla against fractional flow reserve for the detection of flow-limiting coronary heart disease

**DOI:** 10.1186/1532-429X-14-S1-O92

**Published:** 2012-02-01

**Authors:** Roy Jogiya, Geraint Morton, Kalpa De Silva, Simon Redwood, Sebastian Kozerke, Divaka Perera, Eike Nagel, Sven Plein

**Affiliations:** 1 Kings College London, London, UK; 2 LiGHT Institute, Leeds, UK; 3 ETH, Zurich, Switzerland

## Summary

We demonstrate the feasibility of 3D myocardial perfusion CMR at 3 Tesla against fractional flow reserve (FFR) in 53 patients for the detection of flow-limiting coronary artery disease and show good agreement between the techniques. This technique shows excellent diagnostic sensitivity and specificity and may offer an alternative method of detecting ischaemia for the purpose of guiding revascularisation and risk stratification.

## Background

Three-dimensional (3D) myocardial perfusion cardiovascular magnetic resonance (CMR) has recently been proposed to overcome the limited spatial coverage of conventional perfusion CMR methods1. The method has shown good diagnostic accuracy for the detection of coronary artery disease determined by quantitative coronary angiography (QCA)2. However the relationship between the severity of a coronary stenosis on QCA and its functional significance is variable. Pressure wire-derived fractional flow reserve (FFR) <0.75 correlates closely with objective evidence of reversible ischemia and it has been demonstrated that ischaemia-guided PCI confers a prognostic benefit.

## Aim

To determine the diagnostic accuracy of whole heart 3D myocardial perfusion CMR against invasively determined FFR.

## Methods

Fifty-five patients referred for angiography underwent rest and adenosine stress 3D myocardial perfusion CMR at 3Tesla (3D turbo gradient echo, flip angle 15, TR 2.0ms/TE 1.0ms, 12 slices of 5mm thickness, in-plane resolution 2.3x2.3mm2, 10 fold k-space and time k-t broad linear speed up technique acceleration with k-t principal component analysis). Perfusion was scored visually on a patient and coronary territory basis with a score from 0-3. Ischaemic burden was calculated by quantitative segmentation of the volume of hypoenhancement. The FFR was measured in vessels with >50% severity stenosis. Fractional flow reserve <0.75 was considered hemodynamically significant.

## Results

**Figure 1:**
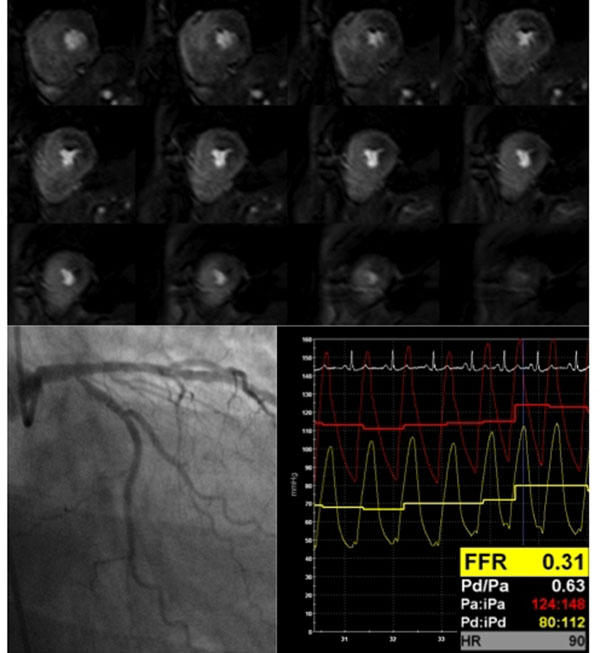
3D CMR perfusion of a patient with a tight ostial circumflex lesion with positive fractional flow reserve (FFR=0.31). Note the transmural perfusion defect from the base towards the apex.

Two patients were excluded (one claustrophobia, on poor image quality). From the remaining fifty-three patients and 159 coronary vessels, 64 underwent pressure wire assessment and 39 had an FFR<0.75 (Figure1). Sensitivity, specificity and diagnostic accuracy of CMR analysis per patient was 90%, 91% and 91%, respectively for the detection of significant coronary artery disease. By coronary territory the values were 79%, 92% and 88%.

## Conclusions

3D CMR stress perfusion can detect functionally significant coronary artery disease with excellent sensitivity, specificity and predictive values when compared with FFR.

3D CMR perfusion imaging may offer an alternative method of detecting ischaemia for the purpose of guiding revascularisation and risk stratification.

## Funding

SP is funded by British Heart Foundation fellowship FS/10/62/28409.

SP/EN receives research grant support from Philips Healthcare.
